# Predicting Climate Change Impacts on the Optimal Habitat of Rare *Potaninia mongolica* Maxim. on the Mongolian Plateau

**DOI:** 10.3390/biology15161347

**Published:** 2026-08-09

**Authors:** Jinting Guo, Wenhui Su

**Affiliations:** 1 College of Life Science and Technology, Inner Mongolia Normal University, Hohhot 010022, China; wenhui_su1017@163.com; 2 Key Laboratory of Biodiversity Conservation and Sustainable Utilization in Mongolian Plateau for College and University of Inner Mongolia Autonomous Region, Hohhot 010022, China

**Keywords:** *Potaninia mongolica* Maxim., climate change, suitable habitat range, MaxEnt model

## Abstract

The combined effects of global climate change and human activities are profoundly reshaping the geographic distribution patterns of rare and endangered plants in arid and semi-arid regions, accelerating habitat fragmentation and population decline, thereby posing a core challenge to biodiversity conservation. As a rare semi shrub endemic to the Mongolian Plateau, *P. mongolica* faces continuous population shrinkage due to habitat fragmentation and climate change caused by extreme weather conditions, overgrazing, and mineral extraction, leaving its survival status critically vulnerable. Based on data from 153 distribution sites, this study employed the MaxEnt model to predict the species’ optimal habitat range, analyzed key environmental factors influencing its distribution, and projected that by the 2050s and 2070s, its total suitable habitat will potentially expand northwestward with increased expansion rates as carbon emissions rise. Its distribution is mainly driven by the minimum temperature of the coldest month, the average temperature in the coldest season and precipitation in the warmest season, which conforms to the law of species movement to the northwest caused by climate warming.

## Simple Summary

*Potaninia mongolica* Maxim. is a rare desert shrub found only on the Mongolian Plateau, but its populations are declining due to climate change and human activities. Using 153 recorded locations and a computer model, we predicted where this plant could survive under future climates. We found that winter temperature and summer rainfall mainly control its distribution. Although the total suitable area may expand northwestward, the best core habitats would shrink under severe warming. This means new areas may become suitable, but the best existing habitats face degradation risks. We suggest prioritizing protection of current core areas in Inner Mongolia and conducting field surveys in new suitable zones before investing in conservation. Our findings support evidence-based conservation decisions for this endangered species.

## 1. Introduction

Global climate change has emerged as one of the most severe challenges facing human society in the 21st century. The Sixth Assessment Report of the Intergovernmental Panel on Climate Change (IPCC) indicates that the global average surface temperature has risen by approximately 1.1 °C compared to pre-industrial levels, with this warming trend continuing unabated. The frequency and intensity of climate events such as extreme heatwaves, droughts, and abnormal precipitation have significantly increased—equivalent to twice the warming rate observed since 1880 [1,2,3]. Arid and semi-arid regions, covering about 36% of the world’s land area, are among the most sensitive areas to climate change and exhibit the most fragile ecosystems. Their inherent vulnerability accelerates and makes ecological degradation irreversible [3,4]. Even minor changes in vegetation patterns or species distribution can trigger dramatic alterations in regional ecosystem structure and function [5,6], exacerbating ecological issues like desertification and biodiversity loss. For rare and endangered plant species with narrow ecological niches, limited dispersal capabilities, and highly specialized habitats, climate-induced changes in habitat suitability, habitat fragmentation, and isolation often lead to sharp population declines, reduced distribution ranges, and even extinction risks [7]. Consequently, investigating how climate change affects the suitable habitats of rare and endangered plants in arid and semi-arid regions has become a central scientific priority in biodiversity conservation.

Species distribution models, also known as ecological niche models (ENMs) or habitat suitability models (HSMs) [8,9,10], are statistical models that utilize observed species distribution data and environmental variables to infer species ecological requirements and map their potential distributions or estimate target species distributions [11,12]. Research on species distribution models originated from studies on the relationship between plant communities and environmental gradients, primarily applied to analyzing species’ response curves to environmental factors [13,14]. The widely used MaxEnt (3.4.4) model offers high computational accuracy, a short processing time, low sample size requirements, and reliable predictions even with limited distribution data [15,16]. Consequently, it is extensively employed in research areas such as pest and disease control [17], habitat prediction for plants and animals [18], and identification of suitable habitats for endangered species along with conservation strategies [19].

*P. mongolica*, a nationally protected second-class wild plant species, forms distinctive desert landscapes in the eastern region of the Afro-Asian desert zone and constitutes a core component of the vegetation on the Alxa Plateau, playing an irreplaceable role in maintaining ecosystem stability in extreme arid areas [20,21]. As the sole genus within the Rosaceae family with three sepals, petals, bracts, and stamens making it the only plant with this characteristic floral structure—it is commonly referred to as the “three-petaled rose” [21,22]. This species primarily inhabits transitional zones between temperate and arid desert regions and warm temperate arid desert areas [23], thriving in gravelly obi soils covered with thin sand layers [24]. Its soil types are predominantly gray or gray-brown desert soils with a pH of approximately 9.0, indicating a preference for slightly alkaline environments [25]. It also occurs in rocky lowlands, gullies along alluvial fans, drainage channels, and saline areas at lake margins [26]. This species is a xerophytic deciduous subshrub, with a plant height of 20–50 cm, developed root system, main root depth of more than 2 m, and strong lateral root horizontal extension ability, which is conducive to absorbing deep soil moisture under extreme drought conditions. Water-adapted ecotypes consist mainly of xerophytic and strongly xerophytic plants, accounting for 60% of its species diversity [22], with concentrated distributions in northeastern Alxa League and northwestern Bayannur City in China, as well as extensive populations in Mongolia’s East Gobi Province and South Gobi Province [21]. Therefore, his study adds a new distribution point of *P. mongolica* in the Mongolian region, rather than being limited to China. This expanded spatial coverage significantly reduces sampling bias and can more accurately characterize the complete niche of species. Firstly, we used the latest coupled model to compare the climate scenarios of the sixth phase of the plan (CMIP6) (SSP1-2.6, SSP2-4.5 and SSP5-8.5). Compared with the older CMIP5 scenarios used in previous studies, CMIP6 can better characterize future radiation forcing and precipitation patterns, thus providing more reliable predictions for conservation planning. Secondly, centroid migration is used to identify dynamic spatial trajectory and migration rate of the range, which is crucial for the design of adaptive protected area networks. Overall, these enable us to test whether incorporating Mongolian data and updating climate models will change the predicted response of the species to warming and aridification and pinpoint areas where conservation interventions should be prioritized in the context of accelerated climate change.

## 2. Data Processing and Research Methods

### 2.1. Data Sources

#### 2.1.1. Data Sources for *P. mongolica* Distribution Points

*P. mongolica* geographical distribution data from the current Etuoke Banner and Hangjin Banner in Ordos City, Inner Mongolia Autonomous Region, Alashan Left Banner and Alashan Right Banner in Alashan League, Urad Rear Banner and Urad Middle Banner in Bayannur City, Wuwei City and Zhangye City in Gansu Province, and South Gobi Province and East Gobi Province in Mongolia were obtained from the published literature and the distribution points recorded by the Chinese Digital Herbarium (CVH, https://www.cvh.ac.cn) and iNaturalist (https://www.inaturalist.org) platform, as well as GBIF Occurrence (https://doi.org/10.15468/dl.arvamh, (accessed on 3 August 2026)). Inaccurate longitude and latitude points were deleted and 164 distribution points were obtained. The points were imported into the ENMTools package of R language to delete the repeated points, and then ArcGIS10.8 was used to eliminate the points outside the study area. Finally, 153 effective distribution data points were obtained.

#### 2.1.2. Sources of Geographical and Environmental Data

The meteorological data were obtained from WorldClim (http://www.worldclim.org), derived from monthly average values interpolated from global weather station records spanning the 30-year period from 1970 to 2000, with a resolution of 30″. The boundary of the study area was downloaded from the standard map service website of the National Bureau of Surveying, Mapping and Geoinformation (http://bzdt.ch.mnr.gov.cn), and the map review number is GS (2019) 1816. Based on the study area of *P. mongolica*, 19 bioclimatic variables (Bio1–Bio19) and three topographic environmental factors were extracted from the raster layer using ArcGIS 10.8 software.

### 2.2. Data Processing

#### 2.2.1. Screening of *P. mongolica* Distribution Sites

This study employed the ENMTools (1.0.4) software package in R to perform duplicate sample screening and cleaning on field distribution point data of *P. mongolica*, aiming to mitigate risks of spatial autocorrelation and model overfitting [27]. First, the original distribution coordinate data were preprocessed by removing invalid records with missing latitude/longitude values or abnormal coordinate deviations. The Trim Duplicata Occurrences tool in the ENMTools package was used to eliminate spatial duplicates, identifying and eliminating multiple duplicate distribution records within the same raster cell while retaining only valid point information, thereby effectively removing spatial duplicates arising from field sampling repetition, coordinate recording errors, and data integration. Finally, the coordinate points along with the study area were loaded into ArcGIS v10.8, where points outside the study area were excluded, yielding a final dataset of 153 *P. mongolica* distribution points.

#### 2.2.2. Processing of Environmental Data

In this study, three shared socio-economic paths (SSP1-2.6, SSP2-4.5 and SSP5-8.5) were selected to represent low-, medium- and high-greenhouse-gas-emission scenarios, respectively. The future climate data are averaged over a 20-year period, with the 2050s corresponding to 2041–2060 and the 2070s corresponding to 2061–2080 [28]. The CMIP6 data uses the Delta downscaling method of the WorldClim standard for downscaling and bias correction. The method uses WorldClim 2.1. (1970–2000) as the baseline climate. Firstly, the climate variable changes between the baseline period and the target period of each GCM were calculated (absolute difference in temperature and relative difference in precipitation), and then the changes were interpolated to a resolution of 30 arcseconds (~1 km) and superimposed on the baseline climate data. All downloaded GeoTiff format raster data were preprocessed by ArcGIS v10.8. Firstly, all layers were converted into the GCS_WGS_1984 geographic coordinate system, and Albers equal area projection was used for projection conversion. In order to ensure spatial matching between the layers of each environmental variable, the Nearest Neighbor method was used to resample all grids to a uniform spatial resolution of 30″ (~1 km). The resampled layers were extracted by the Extract by Mask tool of ArcGIS 10.8 according to the boundary of the study area, ensuring that all the environmental variables entered into the MaxEnt (3.4.4) model had the same spatial range and pixel size. The suitable habitat range of *P. mongolica* was classified into four zones—unsuitable habitat (0.00–0.06), poorly suitable habitat (0.06–0.23), moderately suitable habitat (0.23–0.47), and highly suitable habitat (0.47–0.88)—based on the natural discontinuity breakpoint method [29], and its mapping analysis, spatial transformation, and distribution patterns were conducted using dedicated software tools. The DEM data were derived from NASA Earth data SRTM-30 m. Slope and Aspect were calculated based on the DEM grid through the Slope and Aspect functions in the Surface tool of ArcGIS 10.8. The slope is presented in degrees (°), and the north of the slope is 0° in clockwise rotation.

### 2.3. Research Methods

The ENMTools software toolkit was employed for correlation analysis to screen environmental factors, followed by the MaxEnt (3.4.4) model to calculate the relative contribution rates of environmental variables to species distribution. The jackknife test was then applied to examine the impact of each environmental factor on the potential habitat distribution of *P. mongolica* [30]. All factors were included in the model, and the jackknife test was used to assess their contributions and Importance of Replacement to model construction, with environmental variables contributing minimally or exhibiting low importance values being excluded [31]. The results of the correlation matrix were combined with the contribution rate obtained by the initial operation of 19 climate factors using MaxEnt (3.4.4) to determine the screening threshold. The |R| > 0.85 threshold was used to identify highly correlated variable groups on the correlation matrix and eliminate redundant variables, so as to ensure that the model input variables were representative, independent and interpretable [32]; variables exceeding this threshold were deemed highly correlated. Highly correlated variable pairs were identified, and within each group, only the variable with higher contribution was retained after comparison with others; remaining variables were excluded. This process was repeated once, resulting in a set of uncollinear environmental variables suitable for inclusion in the model.

#### 2.3.1. Construction of MaxEnt (3.4.4)

The nine environmental factor variables were imported along with altitude, slope, aspect, and latitude/longitude data of *P. mongolica* into MaxEnt (3.4.4). Both “Create response curves” and “Use jackknife test to measure variable importance” were checked. In the Basic settings interface, “Random seed” was selected, and 25% of the sampling points were designated as the test dataset and 75% as the training dataset. The bootstrap repeated sampling method was used to run the model, and a total of 10 repeated experiments were carried out. The results were output with an average value of 10 runs. The output format was selected as Logistic type [33], and its accuracy was evaluated based on the AUC value of the ROC curve.

#### 2.3.2. Model Accuracy Evaluation

The AUC value is a metric among various evaluation indicators that is independent of specific thresholds, allowing direct assessment of a model’s overall performance and providing a comprehensive, objective evaluation of its classification ability across all possible thresholds. It also exhibits strong robustness to class imbalance issues, making it widely adopted [34]. Existing research indicates that a higher AUC value (ranging from 0 to 1) corresponds to better model performance [35]: values between 0.5 and 0.6 indicate insufficient model precision and are unsuitable for further analysis; values between 0.6 and 0.7 suggest suboptimal performance, barely acceptable; values between 0.7 and 0.8 represent average performance; values between 0.8 and 0.9 indicate good performance; and values between 0.9 and 1 signify excellent performance [36].

## 3. Research Results

### 3.1. Results of Model Accuracy Evaluation

**Figure 1:**
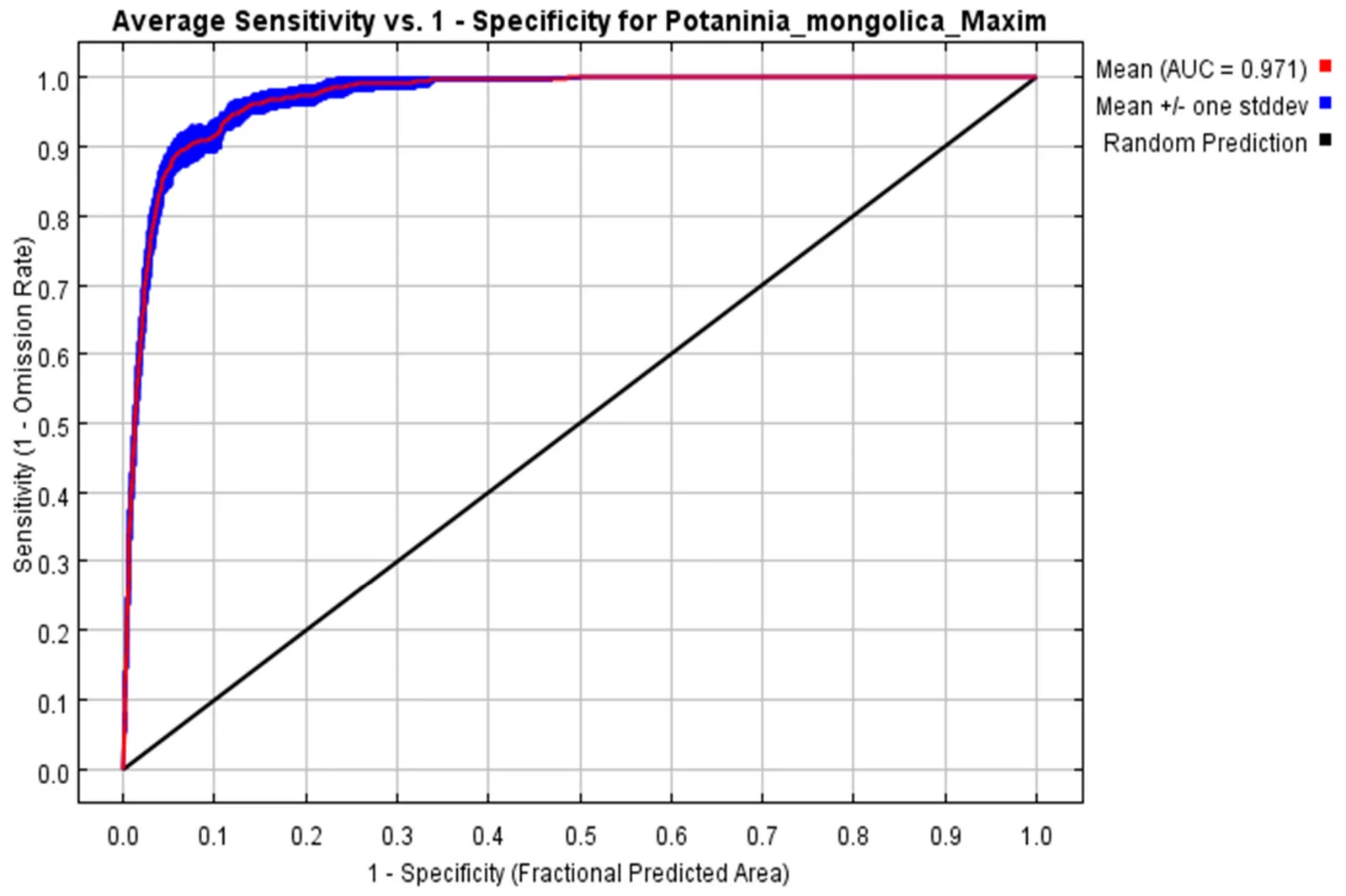
Based on the ROC curve of MaxEnt model on the test set. The ROC curve of 10 repeated bootstrap runs is shown in the figure. The red coarsening curve represents the average of 10 repeated runs, and the blue band represents the mean ± standard deviation. The mean AUC of 10 repeated tests was 0.967 ± 0.019 (range: 0.941–0.984), which was much higher than the random expected value (AUC = 0.5, solid line).

As shown in Figure 1, the AUC value of 0.971 is close to 1, indicating excellent model performance. There is minimal variability across multiple modeling runs, making it suitable for subsequent distribution analysis or conservation planning reference.

### 3.2. Contribution Rate of Environmental Variables

The analysis yielded a correlation coefficient matrix of bioclimatic variables (Table A1). The contribution rate and permutation importance of 19 climatic factors were obtained from the initial operation of MaxEnt (Table A2), resulting in nine final variables: BIO2 (average daily temperature difference), BIO3 (isothermal), BIO5 (maximum temperature in the hottest month), BIO6 (minimum temperature of the coldest month), BIO8 (average temperature in the wettest season), BIO11 (average temperature in the coldest season), BIO12 (average annual precipitation), BIO15 (coefficient of variation of precipitation), and BIO18 (precipitation in the warmest season). Variables bio7 and bio17 were excluded due to their contribution rates being below 1, while bio2 and bio3 were retained as they exhibited low correlation but high substitution importance.

**Figure 2:**
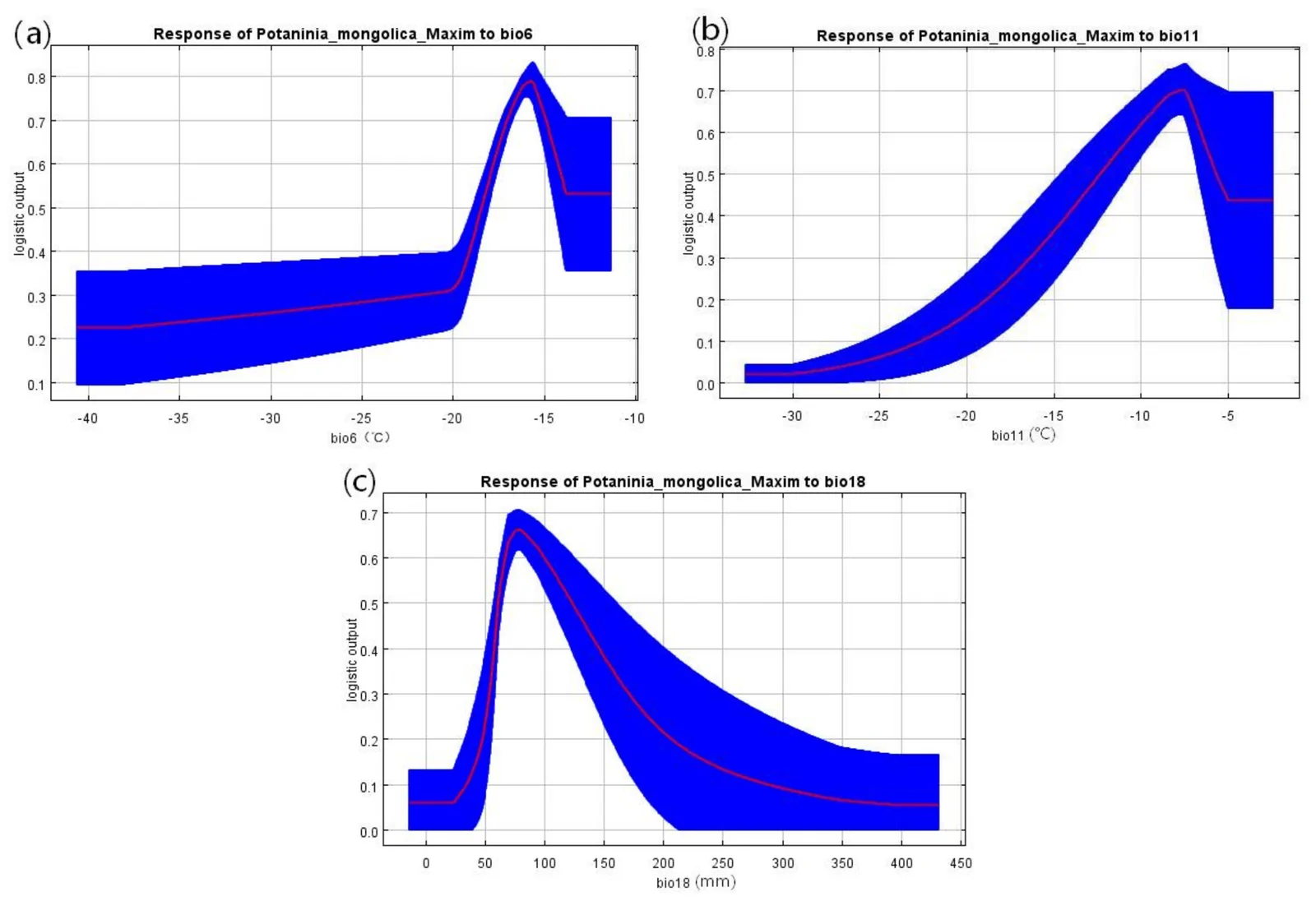
The response curves of three dominant bioclimatic variables to the potential distribution of *P. mongolica*: (**a**) minimum temperature of the coldest month (bio6, °C), (**b**) average temperature in the coldest season (bio11, °C), and (**c**) precipitation in the warmest season (bio18, mm). The red curve represents the average response value of 10 Bootstrap repeated runs, and the blue band represents the mean ± standard deviation, reflecting the uncertainty of the model prediction.

As shown in Table 1, the three dominant environmental factors contributing the most were minimum temperature of the coldest month (bio6), with a contribution rate of 27.4%; average temperature in the coldest season (bio11), with a contribution rate of 25.3%; and precipitation in the warmest season (bio18), with a contribution rate of 21%. The response curve (Figure 2) analysis of the three dominant factors showed that each variable showed a single-peak response pattern—that is, the suitability increased with an increase in the variable value to the optimal value and then decreased, rather than a simple monotonic increase or decrease trend. Specifically, minimum temperature of the coldest month (bio6) increased sharply above about −22 °C, peaked near about −12 °C, and then decreased gradually above −5 °C, indicating that both too low and too high winter temperatures limit the distribution of the species. The suitability of the mean temperature (bio11) in the coldest season increased rapidly above −20 °C, reached the optimum around −10 °C to −5 °C (the suitability was about 0.68–0.70), and gradually decreased after exceeding −5 °C. The suitability of precipitation in the warmest season (bio18) peaked between 40 and 80 mm, and gradually decreased after exceeding 100 mm.

**Figure 3:**
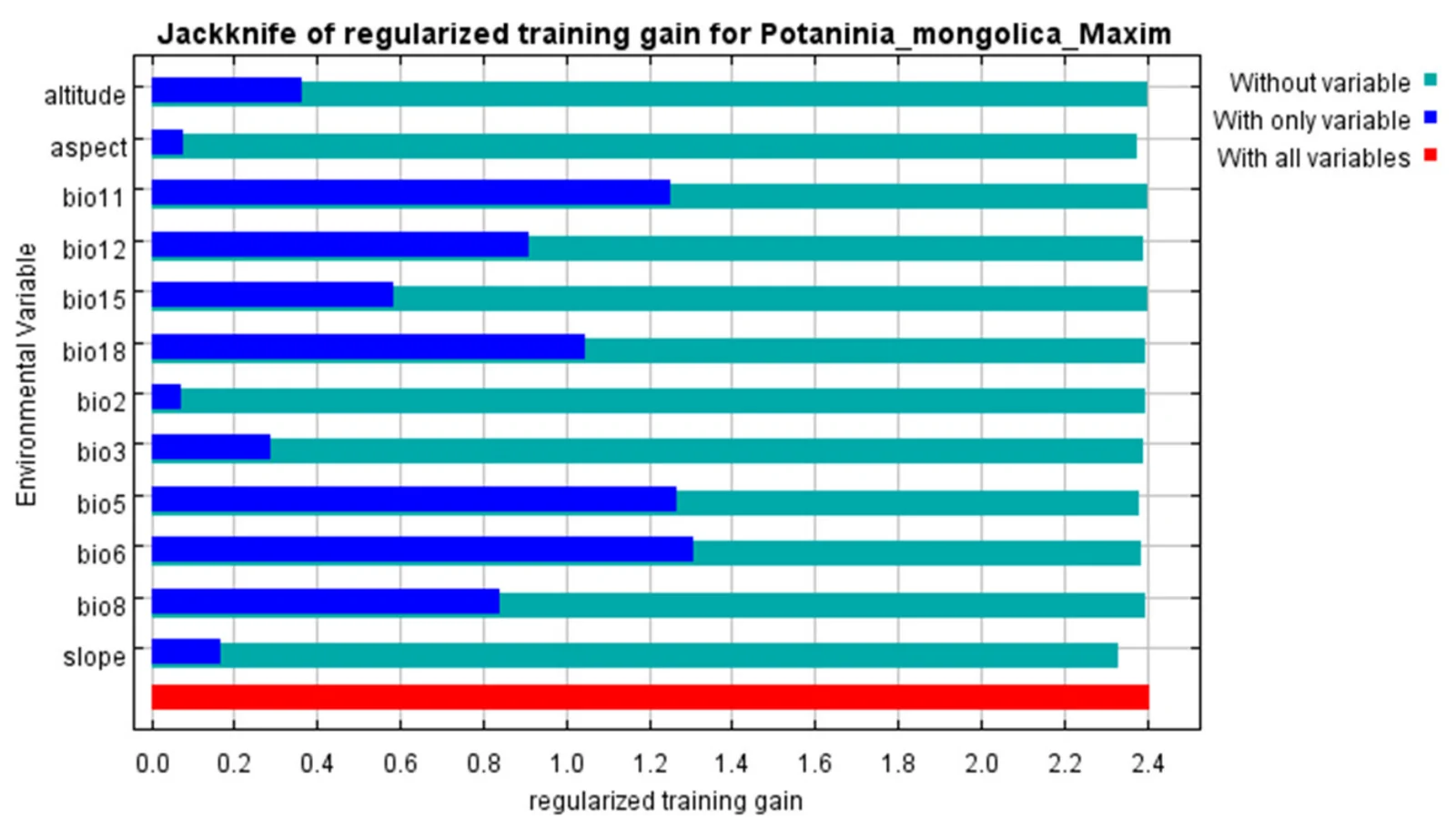
The jackknife test-based regularization training gain test results are used to evaluate the contribution of each environmental variable to the *P. mongolica*. potential distribution model. Variable label control: bio2 = average daily temperature difference, bio3 = isothermal, bio5 = maximum temperature in the hottest month, bio6 = minimum temperature of the coldest month, bio8 = average temperature in the wettest season, bio11 = average temperature in the coldest season, bio12 = average annual precipitation, bio15 = coefficient of variation of precipitation, and bio18 = precipitation in the warmest season.

As shown in Figure 3, minimum temperature of the coldest month (bio6), average temperature in the coldest season (bio11), maximum temperature in the hottest month (bio5), and precipitation in the warmest season (bio18) ranked highest in training gain metrics, making them the dominant environmental factors influencing the geographical distribution of *P. mongolica* under contemporary climate change. The specific values are shown in Table A3.

### 3.3. Potential Suitable Distribution Areas of P. mongolica Under Different Periods and Climate Scenarios

#### 3.3.1. Distribution of Suitable Habitats for *P. mongolica* Under Current Conditions and Climate Scenarios

**Figure 4:**
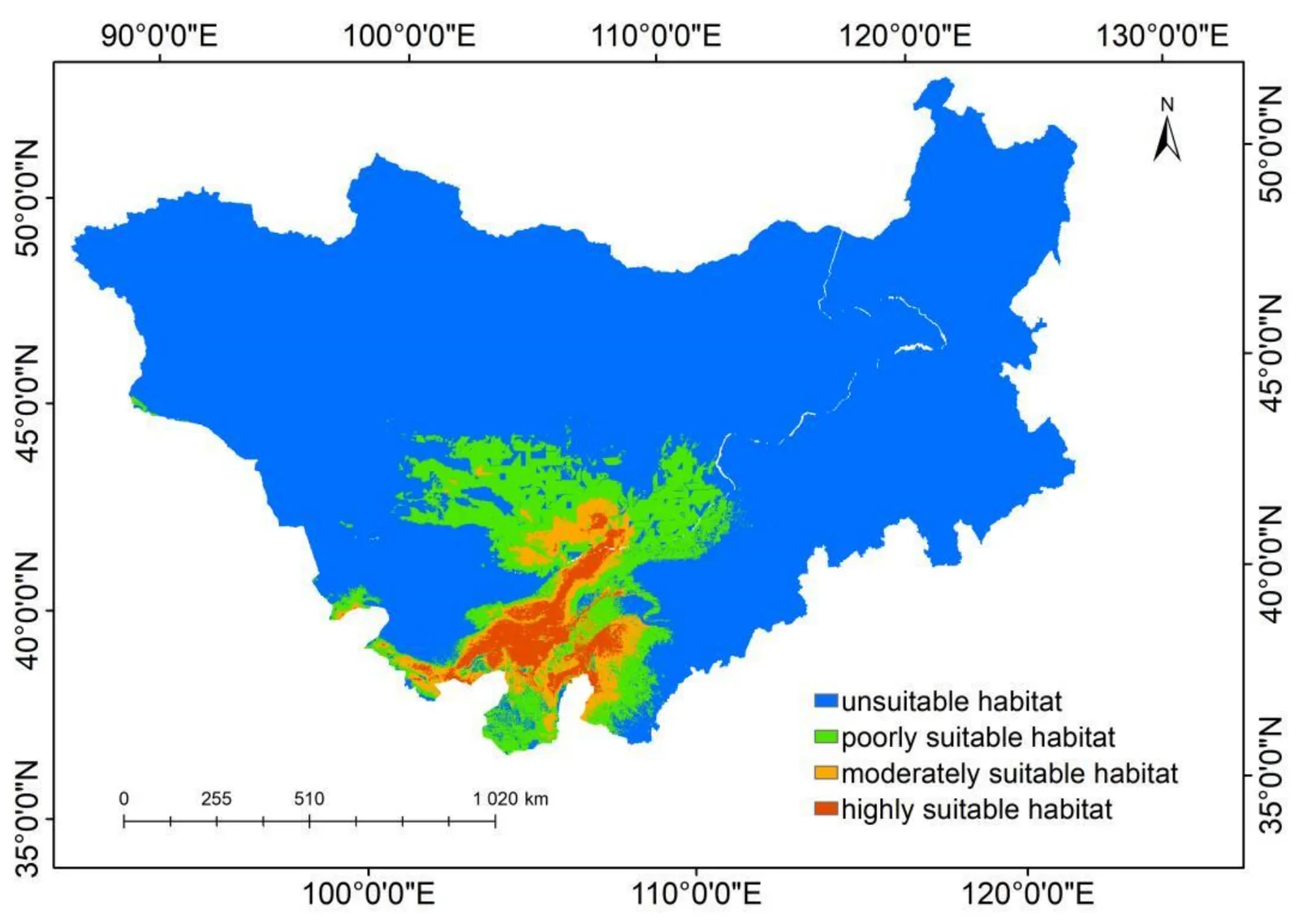
Distribution map of suitable habitats for *P. mongolica* under current climate scenarios.

As shown in Figure 4, blue represents the unsuitable zone, green the lowly suitable zone, yellow the moderately suitable zone, and red the highly suitable zone. Under the current climate scenario, the total area of the suitable zones is 156,700.533314 km^2^, with the poorly suitable habitat covering 80,161.021092 km^2^—primarily distributed across the Southern Gobi, Eastern Gobi, Qianhang’ai, Bayannur City, and Ordos City, with minor distributions in Alxa; the moderately suitable habitat spans 46,096.400238 km^2^, mainly located in the Southern Gobi, Ordos City, and Bayannur City, with limited areas in Alxa; and the highly suitable habitat covers 30,443.111984 km^2^, primarily situated in Alxa and Bayannur City (Table A4).

#### 3.3.2. Distribution of Suitable Habitats for *P. mongolica* Under Future Periods and Climate Scenarios

**Figure 5:**
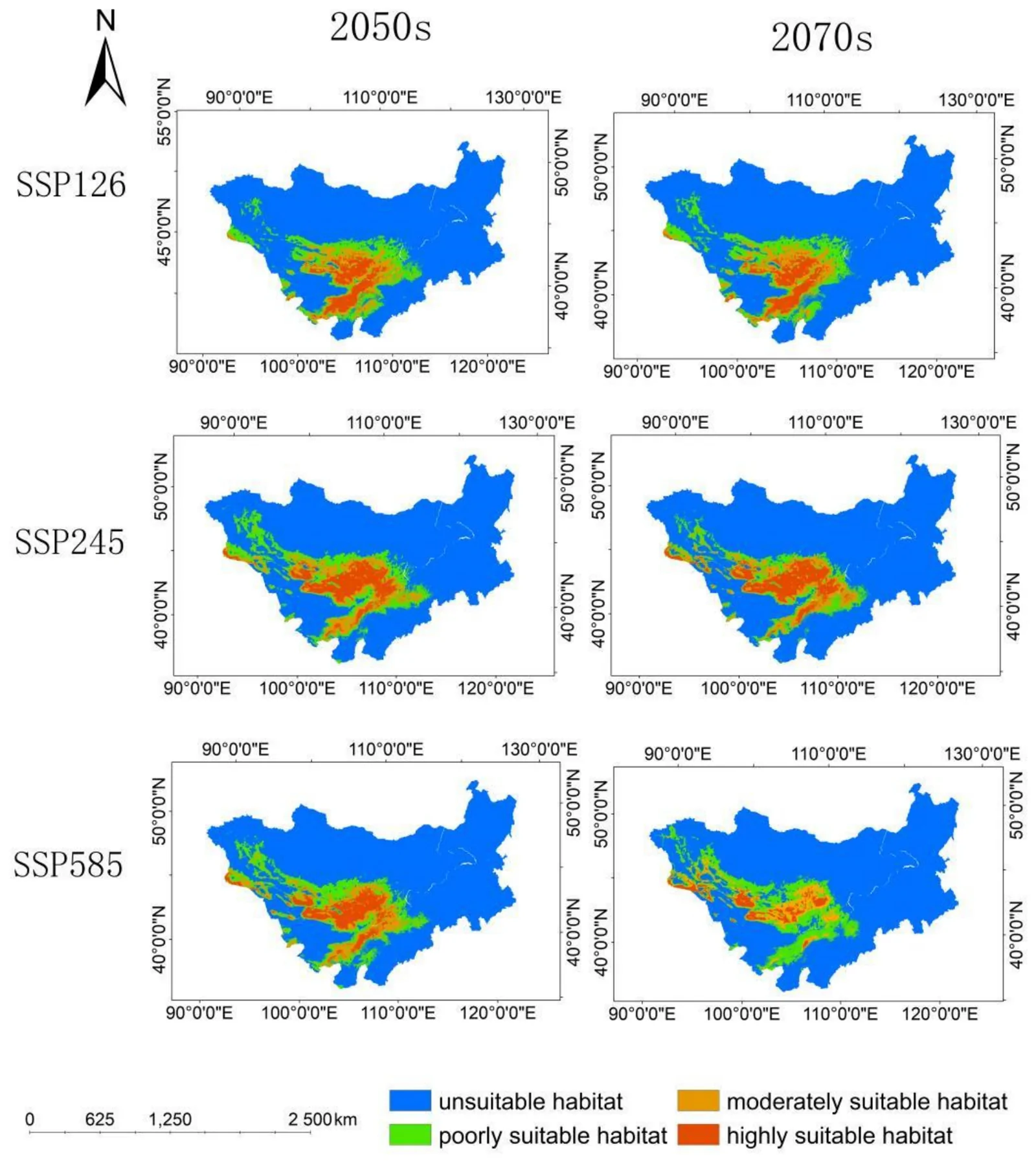
Distribution map of suitable habitats for *P. mongolica* under future climate scenarios.

Figure 5 illustrates the spatial distribution patterns of potential suitable habitats for *P. mongolica* under three typical scenarios (SSP1-2.6, SSP2-4.5, and SSP5-8.5) for the years 2050 and 2070. Blue represents unsuitable habitat, green indicates poorly suitable habitat, yellow denotes moderately suitable habitat, and red signifies highly suitable habitat. Overall, the core suitable habitat of *P. mongolica* remains consistently concentrated in the central–southern part of the study area, specifically in China’s Alxa region. Over time, across all scenarios, the total suitable habitat shows a potential continuous expansion from 2050 to 2070, while the unsuitable habitat correspondingly contracts—particularly the poorly suitable habitat (green). Under SSP5-8.5, the highly suitable habitat shrinks significantly due to excessive warming and drying in the original core region caused by fossil-fuel-intensive conditions with high radiative forcing [37]. In contrast, surrounding cooler regions barely meet moderate suitability thresholds as temperatures rise (with increased proportions of green and orange zones), resulting in a reduction in highly suitable areas compared to SSP2-4.5. The potential expansion of the low-emission scenario (SSP1-2.6) is the gentlest, followed by the medium-emission scenario (SSP2-4.5), and the potential expansion of the high-emission scenario (SSP5-8.5) is the most severe. The suitable area extends farther to the northwest and northeast periphery.

### 3.4. Spatial Variation in the Optimal Habitat Range for P. mongolica Under Different Climate Scenarios

**Figure 6:**
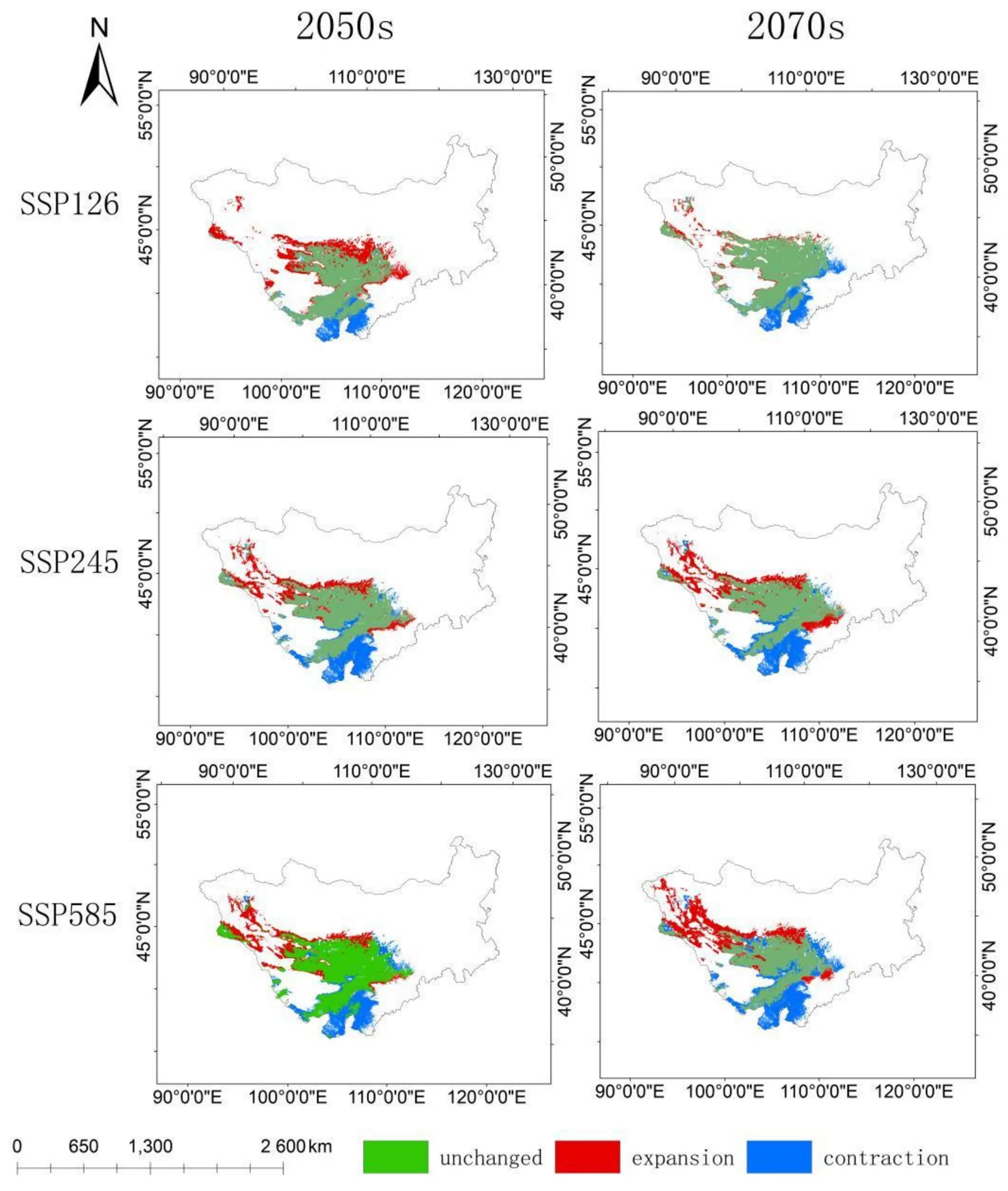
Spatial variation in suitable habitats for *P. mongolica* under different climate scenarios.

Figure 6 illustrates the spatial evolution of *P. mongolica*’s suitable habitats from the 2050s to the 2070s under three climate scenarios: SSP1-2.6 (low emissions), SSP2-4.5 (medium emissions), and SSP5-8.5 (high emissions). Green represents areas that are unchanged, red indicates newly expanded habitats, and blue denotes contracted habitats. Geographically, stable suitable habitats (green) are concentrated in China’s arid northwest region—including traditional distribution zones such as Alxa, Ordos, and Bayannur; expanding habitats (red) extend sporadically along the northwestern and northeastern edges of existing ranges; and contracted habitats (blue) are primarily located at southern margins. As carbon emission intensity increases (from SSP1-2.6 to SSP2-4.5 and then to SSP5-8.5), the red expansion area gradually expands, with the most significant increase observed in the northwest under high-emission scenarios (SSP5-8.5). Southern contraction areas (blue) persist across all scenarios. Over time, compared to the 2050s, all scenarios show a clear pattern of northern expansion and southern contraction: global warming drives species habitats toward the northwest, shifting the overall habitat center northward.

### 3.5. Spatial Pattern Changes in P. mongolica

**Figure 7:**
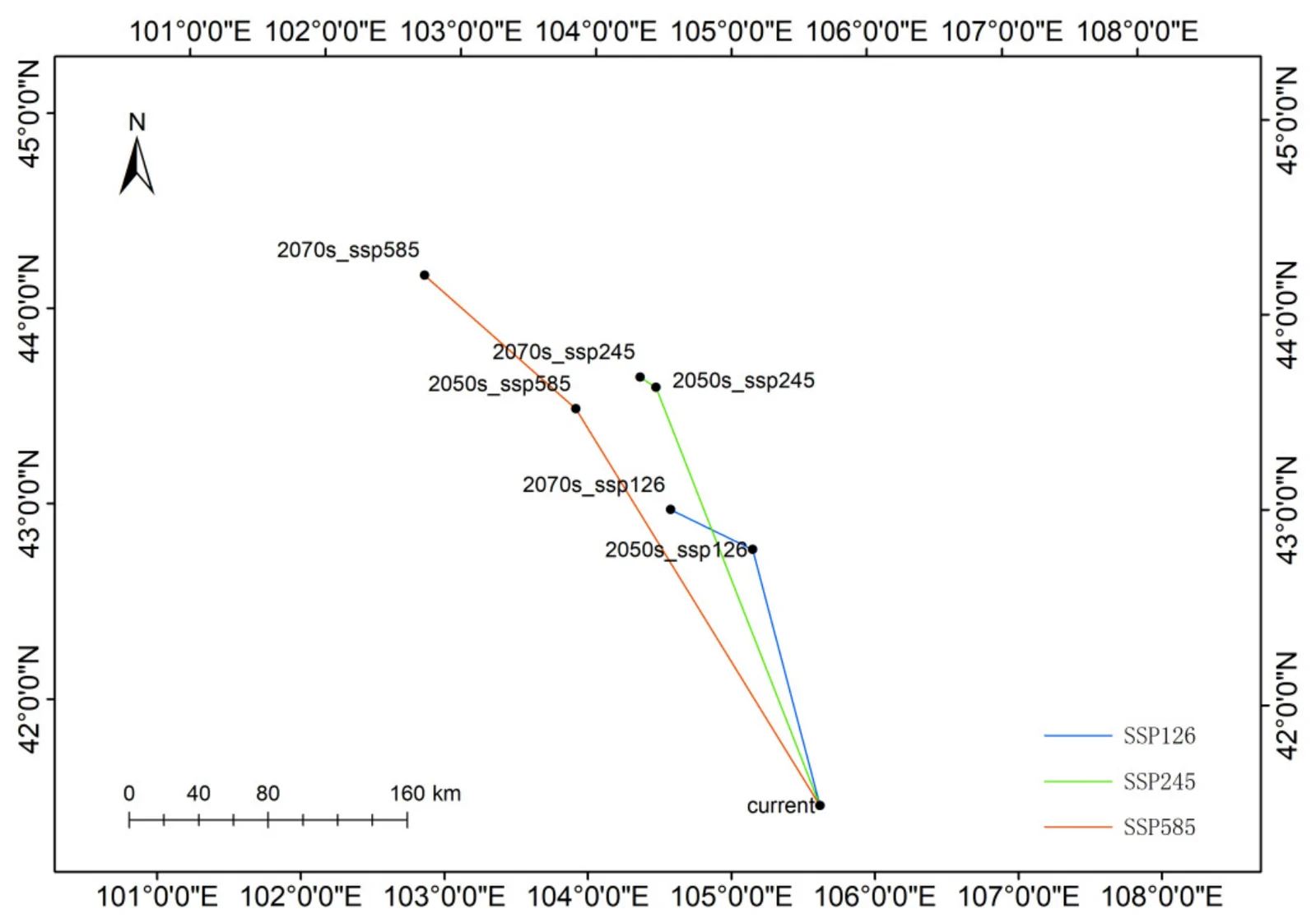
Change in the center of gravity of the optimal habitat range of *P. mongolica* under current and future climate conditions.

The results of the centroid migration analysis (Table 2, Figure 7) show that the centroid of the potential suitable area of *P. mongolica* shows a trend of northward migration under all climate scenarios. Among them, the centroid migration amplitude is the largest under the SSP5-8.5 scenario, where the value for the 2070s relative to the current centroid displacement is about 380.81 km, followed by SSP2-4.5 (267.51 km), and SSP1-2.6 is the smallest (190.82 km). In the meridional (east–west) change, under the low-emission scenario (SSP1-2.6), the center of mass first moves slightly eastward (2050s: 152.35 km, azimuth and then turns northwestward (190.82 km) in the 2070s; however, the medium- and high-emission scenarios (SSP2-4.5 and SSP5-8.5) show continuous northwestward migration, among which the westward movement of SSP5-8.5 is the most significant (the X direction moves from the current 50,999 m to −176,630 m), indicating that the westward movement of suitable habitats in the longitude direction is more prominent under the high-emission scenario. Within the same SSP, the inter-period displacements of the 2070s relative to the 2050s are as follows: SSP1-2.6 about 52.65 km, SSP2-4.5 about 10.90 km, and SSP5-8.5 about 116.20 km. This shows that the high-emission scenario not only has the largest total migration range, but also has the fastest migration speed.

## 4. Discussion

This study found that the minimum temperature of the coldest month (bio6), average temperature in the coldest season (bio11), and precipitation in the warmest season (bio18) are the dominant environmental factors influencing the distribution of *P. mongolica*, accounting for a cumulative contribution rate of 73.7%. This result is consistent with the findings of Qin Yuanyuan et al., who conducted a study on the potential geographical distribution of *P. mongolica* across China based on 73 sampling sites, also identifying rainfall (rainfall in the driest month, annual average rainfall, rainfall in the wettest month, and average rainfall during the warmest quarter) and temperature (maximum temperature in the hottest month) as key environmental variables affecting its distribution [16]. From a biological perspective, *P. mongolica* employs a strategy of “sensitive response and evasive dormancy” to cope with extreme drought, often surviving adverse conditions through a state of “quasi-death” and initiating high metabolic activity when water conditions are favorable. Under drought conditions from July to August, it rapidly enters dormancy to minimize transpiration [38], a survival strategy that results in an extremely narrow threshold for responding to changes in precipitation and temperature [25]. Therefore, the optimal niche range of this species can be summarized as follows: minimum temperature of the coldest month (bio6) ≥20 °C, optimum range of average temperature of the coldest quarter from −10 °C to −5 °C, and precipitation in the warmest season (bio18) between 35 mm and 100 mm. In general, the influence mechanism of average temperature in the coldest season (bio11) on the distribution of *P. mongolica* was similar to that of minimum temperature of the coldest month (bio6), which jointly defined the survival boundary of this species under low-temperature conditions in winter. It should be pointed out that the response curves in Figure 2 show a wide confidence interval at the extreme end of the variable, reflecting the large uncertainty of the suitability estimation under extreme environmental conditions. This may be due to the limited number of distribution records in the marginal habitat, which should be considered when interpreting the predicted distribution boundary.

Qin et al. modeled only based on 73 distribution points in China, and their prediction range was limited to China. In this study, 153 distribution points were integrated, and the distribution records of Mongolia’s South Gobi Province and East Gobi Province were included in the model for the first time, which expanded the niche description of species from ‘partial niche within China’ to ‘complete niche within the Mongolian Plateau’. This expansion is crucial for correctly assessing the impact of climate change on species distributed across borders, because the distribution boundaries and core areas of species may cross national boundaries, and only using local data may lead to erroneous estimates of niche boundaries. In this study, the latest CMIP6 climate scenario was used to predict the climate in arid areas with higher accuracy. However, it should be noted that this study only used a single GCM (IPSL-CM6A-LR), and multi-GCM ensemble analysis is still needed in the future to reduce structural uncertainty.

After incorporating the distribution points of Mongolia’s southern and eastern Gobi provinces into the model, this study found an important phenomenon: the potential suitable area of *P. mongolica* is wider than previously recognized, and its core suitable area includes not only Alxa League and Bayannur City in Inner Mongolia, China, but also extends to the Gobi region in southern Mongolia. However, the distribution records of the Gobi region in southern Mongolia may also be distributed near the water source or the edge of the ancient riverbed, rather than only in the desert hinterland. This suggests that the actual distribution of *P. mongolica* is not only controlled by climatic factors, but also locally regulated by microtopography, soil texture and hydrological conditions. However, the model of this study did not include any soil property variables (such as soil pH, organic matter content, texture, salt content, etc.). This deficiency may lead to bias in the model; land use change and human activities are also not included in the model, and mining activities, grazing pressure, road construction and urbanization of *P. mongolica* habitat fragmentation may, in reality, exceed the direct impact of climate change. Future research needs to integrate high-resolution soil data (such as the SoilGrids global soil database) and a human footprint index to build a more comprehensive prediction model.

The center-of-gravity migration analysis in this study reveals that under various climate scenarios, the optimal habitat range of *P. mongolica* exhibits a predominantly northward and westward migration trend, confirming the pattern whereby climate warming drives species to expand their suitable habitats toward higher latitudes [39]. Under the 2070s_SSP585 scenario, the center of gravity migrated northward by 380.8100859 km. This spatial pattern change aligns with the response patterns observed for most rare and endangered plant species to climate change. Existing research indicates that climate change fundamentally alters the distribution patterns of rare and endangered species; approximately 31% of species, including *Dipteronia sinensis* Oliv. and *Picea brachytyla* (Franch.) *E. Pritz.*, demonstrate a clear northward migration trend [40]. The suitable habitat area of *Amygdalus mongolica* showed a decreasing trend under SSP1-2.6, SSP3-7.0 and SSP5-8.5 scenarios, and the centroid moved to high latitudes overall [41]. However, *Allium mongolicum* showed a trend of northward expansion similar to *A. mongolicum* [42]. The underlying mechanism of this migration lies in climate warming exceeding the temperature thresholds of species’ original habitats, compelling them to relocate to higher latitudes (northern regions) and elevations in search of suitable ecological niches [43]. As a strongly drought-tolerant plant native to arid desert regions [44], *P. mongolica* demonstrates certain drought resistance but has limited tolerance to extreme heat.

This study reveals a phenomenon that needs special attention: the total suitable area shows a net increase, but the highly suitable area shrinks significantly under the high-emission scenario. These two trends are not contradictory, but reflect different ecological processes. The expansion of the total suitable area occurs because climate warming has made the climatic conditions in the northern marginal areas (southern Mongolia and northern Inner Mongolia, China) reach the survival threshold of *P. mongolica*, creating a new potential suitable environment in space. The shrinkage of highly suitable habitats occurs because the original core area exceeds the optimal niche threshold of *P. mongolica* due to excessive warming and drought, resulting in the loss of core high-quality habitats. The total expansion suggests potential opportunities for assisted migration and natural diffusion, but the newly added areas were mainly poorly suitable areas and medium suitable areas. Whether the habitat quality in these areas is sufficient to maintain viable populations is still uncertain. Shrinkage of highly suitable areas warns of the risk of degradation of the core population—even if the total suitable area increases, the optimal living environment of the species is still shrinking. Therefore, the protection strategy should not be misled by the increase in the total suitable area. First of all, strict in situ protection should be given as a priority to the existing highly suitable areas to prevent the further loss of the core population. At present, the core suitable area of *P. mongolica* is also concentrated in Alxa League and Bayannaoer City, Inner Mongolia, China, which should be the core position for long-term protection [45]. Secondly, for areas predicted to be new suitable areas, targeted field surveys should be carried out to confirm the actual existence of species and habitat quality, rather than assuming that species will colonize automatically. However, the northward and westward movement of the suitable areas in the future will lead to a large number of new suitable areas outside the existing protected area system, forming obvious protection vacancies and increasing protection positions.

However, from the perspective of protection practice, it is necessary to further clarify the minimum acceptable standards and optimal management solutions to guide the effective allocation of protection resources. It is prohibited to carry out new mining rights approval and mineral resource exploration activities in the core suitable areas; the core suitable area is included in ecological protection red line management, limiting grazing intensity and controlling livestock carrying capacity. A regular monitoring system should be established for core populations, and systematic surveys of population size and habitat quality should be conducted at least every 3 years. Under the premise that the minimum standards are met, it is recommended to further implement the following measures to maximize the long-term survival probability and genetic diversity maintenance capacity of *A. mongolica*. A dynamic protection network based on climate prediction should be implemented. In view of the uncertainty of future climate change, it is recommended to adopt ‘dynamic conservation planning’. 

The new suitable areas predicted in this study (especially in the northwestern part of South Gobi Province in Mongolia and northern Inner Mongolia in China) are potential areas identified under the assumption of unrestricted diffusion, ignoring soil heterogeneity and human disturbance. However, potential suitability does not mean actual occupancy. Before putting conservation resources into these areas, systematic field surveys must be carried out to verify the predicted results. Specifically, future verification work should focus on (1) whether there are existing populations or historical distribution records of *P. mongolica* in the target area; (2) whether the soil type and pH value meet the substrate preference of the species; (3) whether the land use status (grazing intensity, mining activities, etc.) allows the population to survive; and (4) whether habitat connectivity is sufficient to support gene flow and natural regeneration.

This study has obvious deficiencies in the quantification of predictive uncertainty, which needs special attention when interpreting the results. First of all, this study did not perform MESS (multivariate environmental similarity surface) analysis and clamp map analysis. MESS analysis is a key tool to identify whether there is a combination of environmental conditions outside the scope of training data in future predictions. The lack of this analysis means that we cannot clearly determine which prediction areas are interpolation (high reliability) and which are extrapolation (low reliability). Secondly, this study did not quantify MaxEnt parametric uncertainty (such as a CV map based on bootstrap repeated experiments) and GCM structural uncertainty (such as a multi-model standard deviation map). Therefore, the consensus suitability map presented in this study is not accompanied by a supporting uncertainty layer, which may make readers overestimate the certainty of the prediction results. We suggest that when referring to the prediction results of this study, readers should focus on the stability judgment of the core suitable areas (Alashan League and Bayannur City), and the prediction of the new suitable areas in the northwest should be regarded as exploratory. Future research should give priority to supplementary MESS analysis and multi-GCM set uncertainty assessment to improve the reliability of prediction results.

## 5. Conclusions

This study employed the MaxEnt model to systematically analyze the habitat distribution patterns of *P. mongolica*, a rare and endangered plant species on the Mongolian Plateau, as well as its response to future climate change.

Under current climatic conditions, the total optimal distribution area of *P. mongolica* spans 156,700.533314 km^2^. The core highly suitable zone is concentrated in Alxa League and Bayannur City of Inner Mongolia, China, while the moderately and mildly suitable zones extend to South Gobi Province and East Gobi Province in Mongolia, as well as Ordos City in China.

In the 2050s and 2070s, under all three shared socioeconomic scenarios, the total suitable habitat range of *P. mongolica* exhibits a sustained potential expansion trend. The habitat range shifts overall toward the northwest, forming a spatial pattern characterized by “expansion in the north and contraction in the south,” while its center of gravity moves toward the northwest. Among these factors, minimum temperature of the coldest month (bio6), average temperature in the coldest season (bio11), and precipitation in the warmest season (bio18) are the dominant environmental determinants influencing *P. mongolica*’s distribution.

## Figures and Tables

**Table 1:** Contribution percentages and substitution importance of impact factors for *P. mongolica*.

Order Number	Variable	Contribution Percentage	Importance of Replacement
1	bio6	27.4	11.6
2	bio11	25.3	12.4
3	bio18	21	41.1
4	bio12	8	11.1
5	bio3	6.1	2.9
6	Slope	4.4	7.6
7	bio5	2.5	4.7
8	bio2	1.6	2.8
9	bio8	1.4	2.3
10	Aspect	1.1	1.1
11	bio15	0.9	0.7
12	Altitude	0.2	1.7

**Table 2:** Center coordinates and migration distances of the optimal habitat zone under different scenarios.

Scenario	Longitude	Latitude	X_m	Y_m	Distance from Current (km)	Inter-Period Displacement (km)
current	105.620279	41.538554	50,999.3484	4,473,610.511	——	——
2050s_ssp126	105.152489	42.84854	12,311.13369	4,620,969.577	152.3531167	——
2050s_ssp245	104.456246	43.678552	−43,388.77823	4,714,355.119	258.5867066	——
2050s_ssp585	103.879795	43.564906	−89,529.1425	4,701,979.693	268.1431336	——
2070s_ssp126	104.564312	43.051954	−35,074.67198	4,643,911.417	190.8170213	52.64734206
2070s_ssp245	104.34113	43.730792	−52,535.43607	4,720,275.823	267.5130424	10.89569113
2070s_ssp585	102.768621	44.237149	−176,629.9217	4,778,899.287	380.8100859	116.2031398

(Note: The centroid is calculated based on the weighted average method of suitability (with the Logistic suitability value output by MaxEnt as the weight), covering all the suitable area grids, the projection coordinate system is Albers equal area projection, and the migration distance is calculated based on the projection coordinate: D = √[(ΔX)2 + (ΔY)2]/1000, in km, relative to the current. This indicates the linear displacement of the centroid of the scene relative to the current centroid; the ‘time interval’ represents the centroid displacement of 2070s relative to 2050s in the same SSP.)

## Data Availability

All data is provided in the manuscript.

## Abbreviations

AUC	Area Under the Curve
Bio	Bioclimatic variable
CMIP6	Coupled Model Intercomparison Project Phase 6
CVH	Chinese Virtual Herbarium
DEM	Digital Elevation Model
EHM	Ecological Niche Model
GCM	Environmental Niche Model Tools
GCS	Geographic Coordinate System
GIS	Geographic Information System
HSM	Habitat Suitability Model
IPCC	Intergovernmental Panel on Climate Change
MESS	Multivariate Environmental Similarity Surface
ROC	Receiver Operating Characteristic
SDM	Species Distribution Model
SRTM	Shuttle Radar Topography Mission
SSP	Shared Socioeconomic Pathway

## Appendix A

**Table A1:** This is a correlation matrix table.

**SPECIES**	**Bio19**	**Bio1**	**Bio2**	**Bio3**	**Bio4**	**Bio5**	**Bio6**
bio19	0	−0.436172573	0.15244053	−0.086511259	0.268430191	−0.363142143	−0.480206556
bio1	0	0	−0.061774888	0.184944367	−0.35273279	0.905912638	0.901278806
bio2	0	0	0	0.503440324	0.208327651	0.120795607	−0.24351264
bio3	0	0	0	0	−0.724564306	−0.051100106	0.384507798
bio4	0	0	0	0	0	0.051573655	−0.697537035
bio5	0	0	0	0	0	0	0.65104237
bio6	0	0	0	0	0	0	0
bio7	0	0	0	0	0	0	0
bio8	0	0	0	0	0	0	0
bio9	0	0	0	0	0	0	0
bio10	0	0	0	0	0	0	0
bio11	0	0	0	0	0	0	0
bio12	0	0	0	0	0	0	0
bio13	0	0	0	0	0	0	0
bio14	0	0	0	0	0	0	0
bio15	0	0	0	0	0	0	0
bio16	0	0	0	0	0	0	0
bio17	0	0	0	0	0	0	0
bio18	0	0	0	0	0	0	0
**SPECIES**	**bio7**	**bio8**	**bio9**	**bio10**	**bio11**	**bio12**	**bio13**
bio19	0.257141667	−0.402350818	−0.507735351	−0.377481831	−0.4588116	0.721171238	0.647174202
bio1	−0.255944361	0.908899376	0.913077973	0.931457778	0.926990374	−0.405038498	−0.358335362
bio2	0.438503996	0.001915649	−0.12670322	0.012729999	−0.126914418	0.209894776	0.20451332
bio3	−0.551315218	−0.125647498	0.351308664	−0.083863024	0.43653325	0.01794043	0.016915449
bio4	0.958589229	0.048886458	−0.578699257	0.007727281	−0.675212186	0.203280083	0.199007776
bio5	0.164312274	0.982933705	0.721159251	0.99066897	0.695726668	−0.365621575	−0.321431178
bio6	−0.641750531	0.668130146	0.938953011	0.697690173	0.989323412	−0.426593519	−0.386436986
bio7	0	0.12486265	−0.491573159	0.094263555	−0.582728103	0.184963973	0.177427454
bio8	0	0	0.727639951	0.993374039	0.69921761	−0.367019863	−0.313649252
bio9	0	0	0	0.75366265	0.94950637	−0.483988723	−0.435770921
bio10	0	0	0	0	0.731974459	−0.360313133	−0.310846053
bio11	0	0	0	0	0	−0.399966163	−0.360372472
bio12	0	0	0	0	0	0	0.987390427
bio13	0	0	0	0	0	0	0
bio14	0	0	0	0	0	0	0
bio15	0	0	0	0	0	0	0
bio16	0	0	0	0	0	0	0
bio17	0	0	0	0	0	0	0
bio18	0	0	0	0	0	0	0
**SPECIES**	**bio14**	**bio15**	**bio16**	**bio17**	**bio18**	-	-
bio19	0.946274345	−0.15255464	0.661329824	0.98876352	0.658709396	-	-
bio1	−0.392485337	−0.088058919	−0.398522317	−0.416401932	−0.404381633	-	-
bio2	0.145932299	0.111110054	0.219171022	0.16015975	0.217611216	-	-
bio3	−0.043978233	−0.067028522	0.013897336	−0.041385107	0.008109256	-	-
bio4	0.205626449	0.183724605	0.217302723	0.216142027	0.223257059	-	-
bio5	−0.339236474	−0.016966946	−0.352852827	−0.361802346	−0.356652562	-	-
bio6	−0.418430885	−0.12956736	−0.424742259	−0.444790243	−0.430864821	-	-
bio7	0.201015006	0.15123569	0.195459352	0.212470271	0.199576753	-	-
bio8	−0.375568402	0.012877394	−0.348761766	−0.401398479	−0.350834765	-	-
bio9	−0.470750788	−0.096962665	−0.475429903	−0.488900906	−0.48138074	-	-
bio10	−0.351384322	−0.009185609	−0.346474829	−0.374679146	−0.350192086	-	-
bio11	−0.397855605	−0.132317598	−0.399327746	−0.42186976	−0.406090604	-	-
bio12	0.69646358	0.351210928	0.993494994	0.74193492	0.992667139	-	-
bio13	0.626402115	0.448923586	0.995498178	0.667214795	0.995120201	-	-
bio14	0	−0.136467185	0.639439052	0.957240167	0.636808937	-	-
bio15	0	0	0.441553729	−0.143153985	0.444222665	-	-
bio16	0	0	0	0.681206217	0.999812051	-	-
bio17	0	0	0	0	0.678319717	-	-
bio18	0	0	0	0	0	-	-

**Table A2:** Contribution rate and replacement importance of 19 climate factors.

Variable	Percent Contribution (%)	Permutation Importance (%)
bio6	46.2	33.3
bio12	12	7.4
bio18	11.5	16.1
bio1	8.8	9.1
bio2	3.3	2.7
bio16	3.1	9.6
bio3	3	0.7
bio15	2.4	2.2
bio11	1.7	0.1
bio5	1.7	3.5
bio9	1.6	3.2
bio13	1.5	0.4
bio8	1.1	0.2
bio10	0.8	0.1
bio7	0.7	0
bio17	0.3	0
bio14	0.2	10.4
bio4	0.1	1
bio19	0	0

**Table A3:** Regularized training gain values from the jackknife test for each environmental variable.

Variable Code	Variable Description	With Only Variable	Without Variable	With all Variables
Bio6	Min Temperature of Coldest Month	1.3065	2.3849	2.4085
Bio5	Max Temperature of Warmest Month	1.2652	2.3802	2.4085
Bio11	Mean Temperature of Coldest Quarter	1.255	2.3998	2.4085
Bio18	Precipitation of Warmest Quarter	1.0473	2.3978	2.4085
Bio12	Annual Precipitation	0.9121	2.3905	2.4085
Bio8	Mean Temperature of Wettest Quarter	0.8404	2.3987	2.4085
Bio15	Precipitation Seasonality	0.5834	2.4004	2.4085
Altitude	Elevation	0.3653	2.4027	2.4085
Bio3	Isothermality	0.2899	2.3947	2.4085
Slope	Slope Gradient	0.1695	2.3335	2.4085
Bio2	Mean Diurnal Range	0.0716	2.3983	2.4085
Aspect	Aspect	0.0766	2.3782	2.4085

(Note: The regularized training gain is a dimensionless measure of model fit, with higher values indicating better explanatory power of the model for the presence records.)

**Table A4:** Suitable habitat areas (km^2^) of *P. mongolica* under current and future climate scenarios.

Scenario	Unsuitable Habitat (km^2^)	Porrly Suitable Habitat (km^2^)	Moderately Suitable Habitat (km^2^)	Highly Suitable Habitat (km^2^)
Current	2,540,274.812	80,161.02109	46,096.40024	30,443.11198
2050s_ssp126	2,373,812.718	158,814.4457	90,782.265	73,565.91688
2050s_ssp245	2,318,312.748	167,063.3681	93,508.79604	118,090.4335
2050s_ssp585	2,341,267.039	165,312.489	97,272.74505	93,123.0732
2070s_ssp126	2,392,568.176	148,070.9302	92,997.01999	63,339.21953
2070s_ssp245	2,313,835.338	167,283.9613	105,550.6597	110,305.387
2070s_ssp585	2,438,297.761	138,227.4346	73,327.6763	47,122.47341
